# Supplementary material for: Complexome profiling of the Chlamydomonas psb28 mutant reveals TEF5 as an early PSII assembly factor
Source: Plant Cell. 2025 Mar 17;37(6):koaf055. doi: 10.1093/plcell/koaf055 (PMC12164590; doi:10.1093/plcell/koaf055)
Supplement: koaf055_Supplementary_Data [file koaf055_supplementary_data.zip › psb28_interactive.zip!/plots/intensity/qualityPlots/peptides/FKB20_Cre13.g577850.t1.1.html]

### Cre13.g577850.t1.1

Trivial name: FKB20  
SeqCoverage [median]: 16.60  
Mass: 20.43 kDa  
  
 

| Sequence | MissedCleavage | Mass [Da] | wtIntensity | muIntensity | Score |
| --- | --- | --- | --- | --- | --- |
| IQTQNRVPVDFPGFIR | 1 | 1886.02 | 499.87 | 0.00 | 30.74 |
| VPVDFPGFIR | 0 | 1145.62 | 290.52 | 4009.98 | 82.43 |
|  |  |  | 790.4 | 4010.0 |  |
